# Self-Reported Health, Well-Being, and COVID-19 Vaccination Willingness Across the Generations

**DOI:** 10.1093/geroni/igab046.309

**Published:** 2021-12-17

**Authors:** Taylor Patskanick

**Affiliations:** MIT, Somerville, Massachusetts, United States

## Abstract

The COVID-19 pandemic has impacted the generations’ health and wellbeing across a range of dimensions. In the first survey, the 50+ adopted the smallest number of health behaviors (e.g., handwashing, mask-wearing, etc.) in response to the pandemic compared to younger age groups. In the first and second surveys, the Baby Boomer generation reported less intense worry than other generations, especially regarding their socioemotional health and family members’ health. For younger generations, worries tended to increase from March to June—especially those related to socio-emotional health and COVID-19 in general. In the third survey wave, older generations self-reported better psychological wellbeing, less personal burnout, and better cognitive health compared to younger generations. Willingness to get the COVID-19 vaccine did not vary by generation in this sample; however, implications of this (including additional factors that may be influential such as psychological wellbeing) will be discussed.

